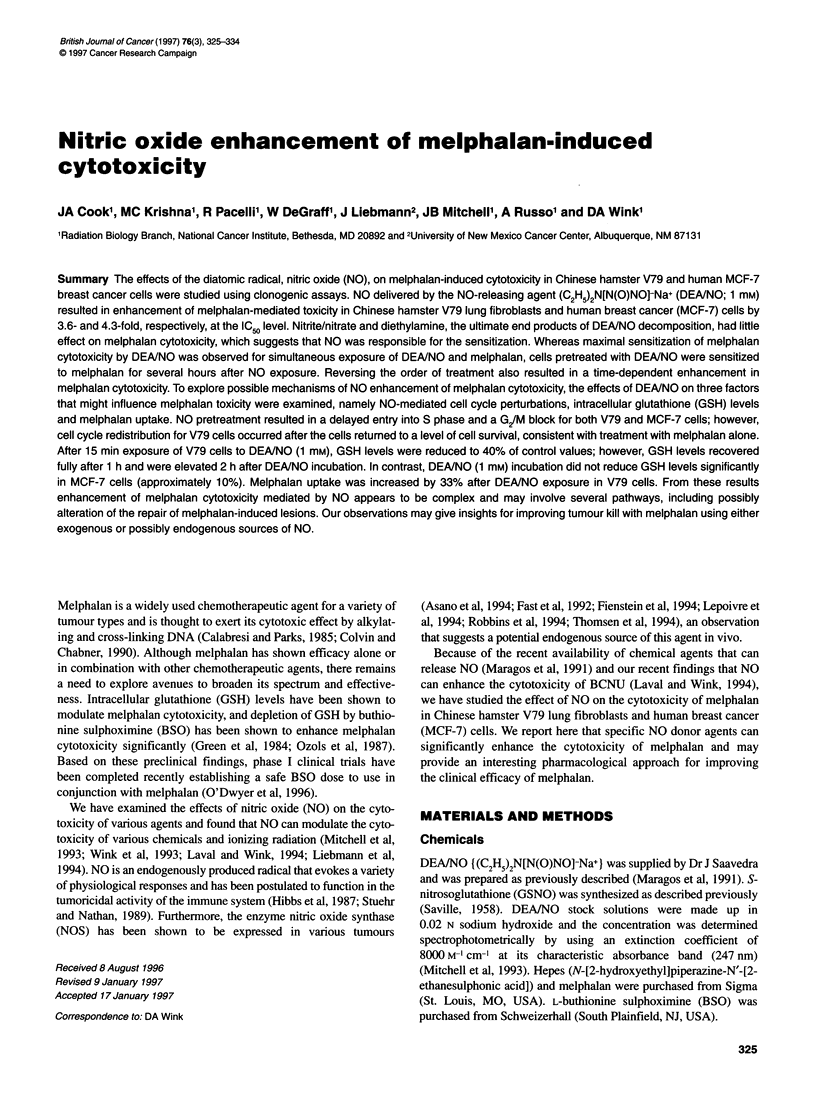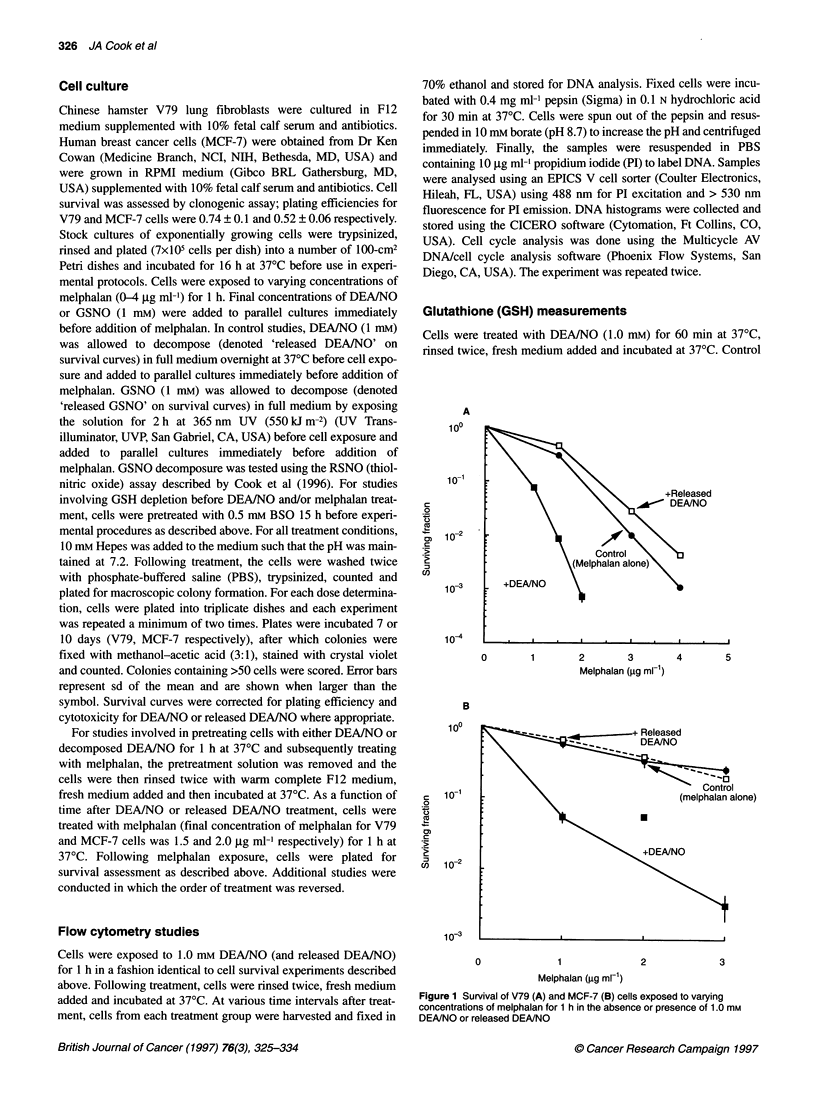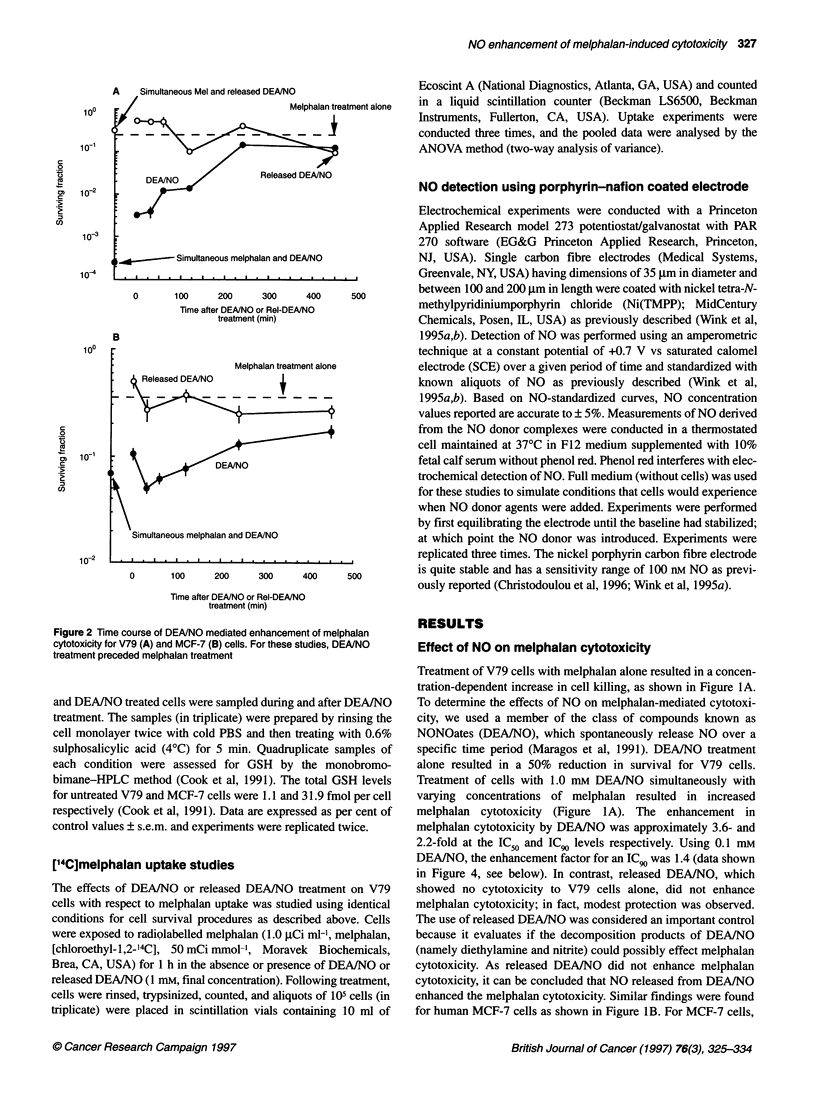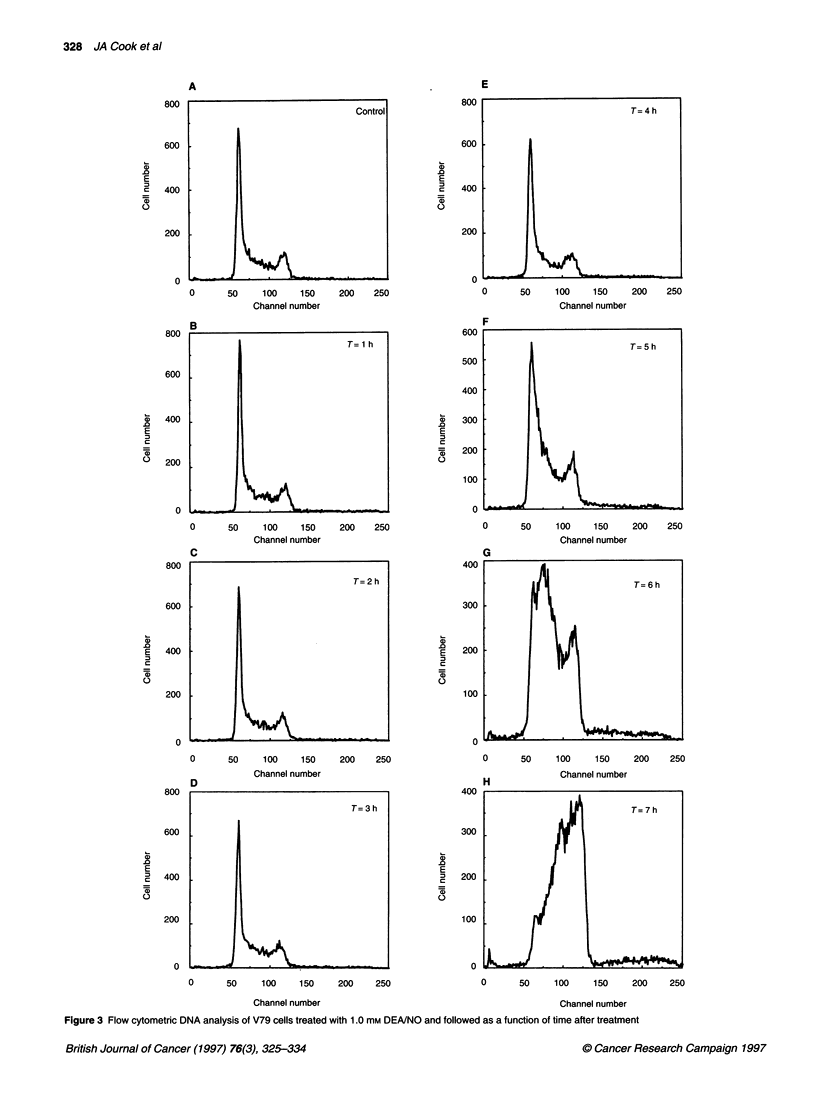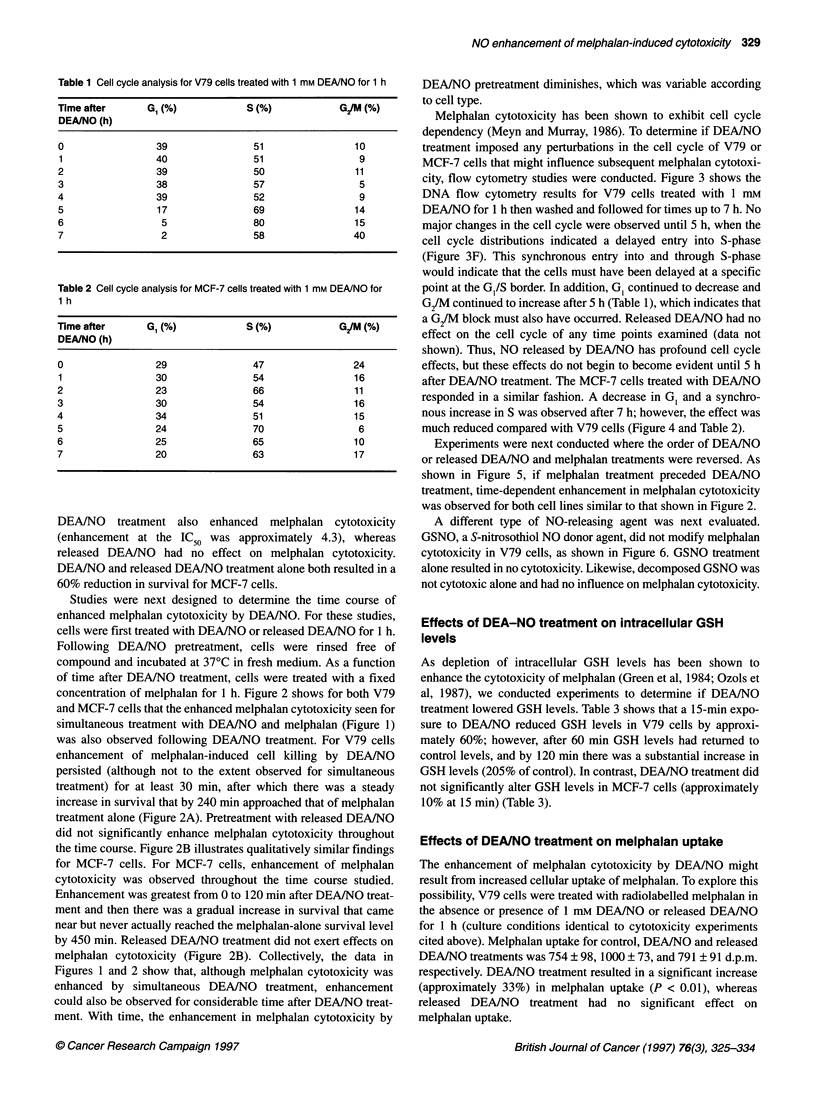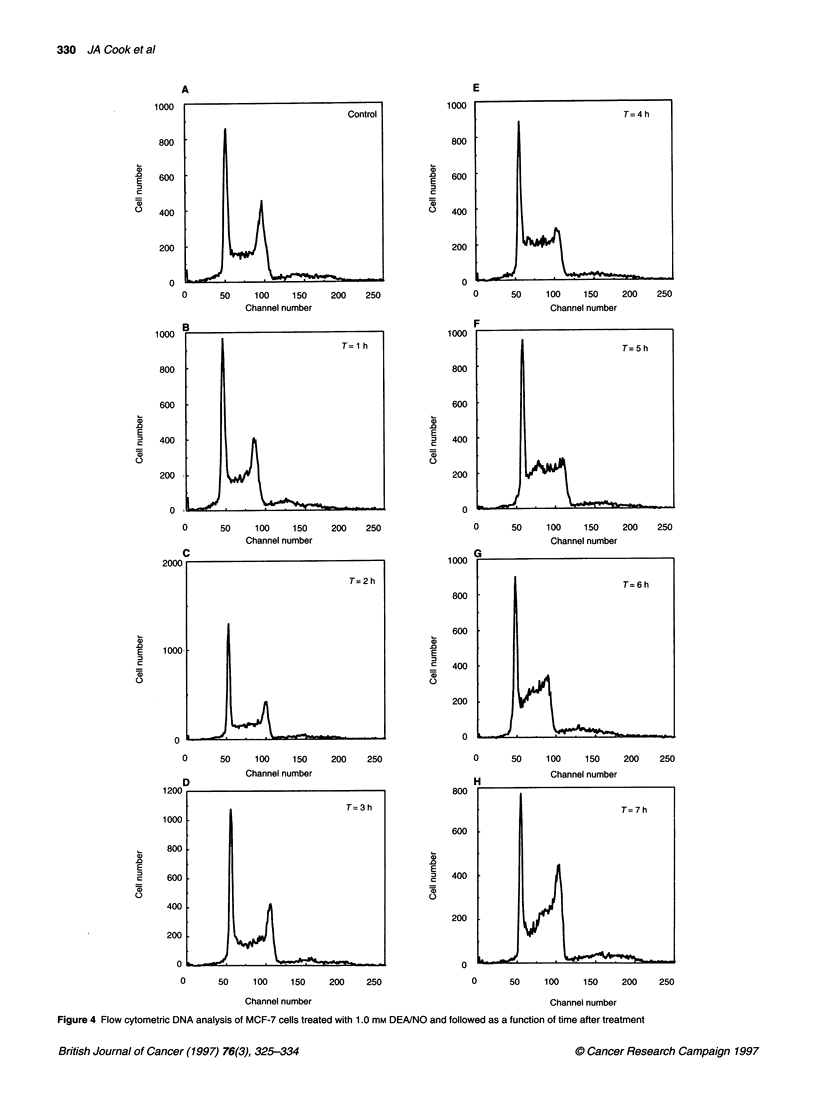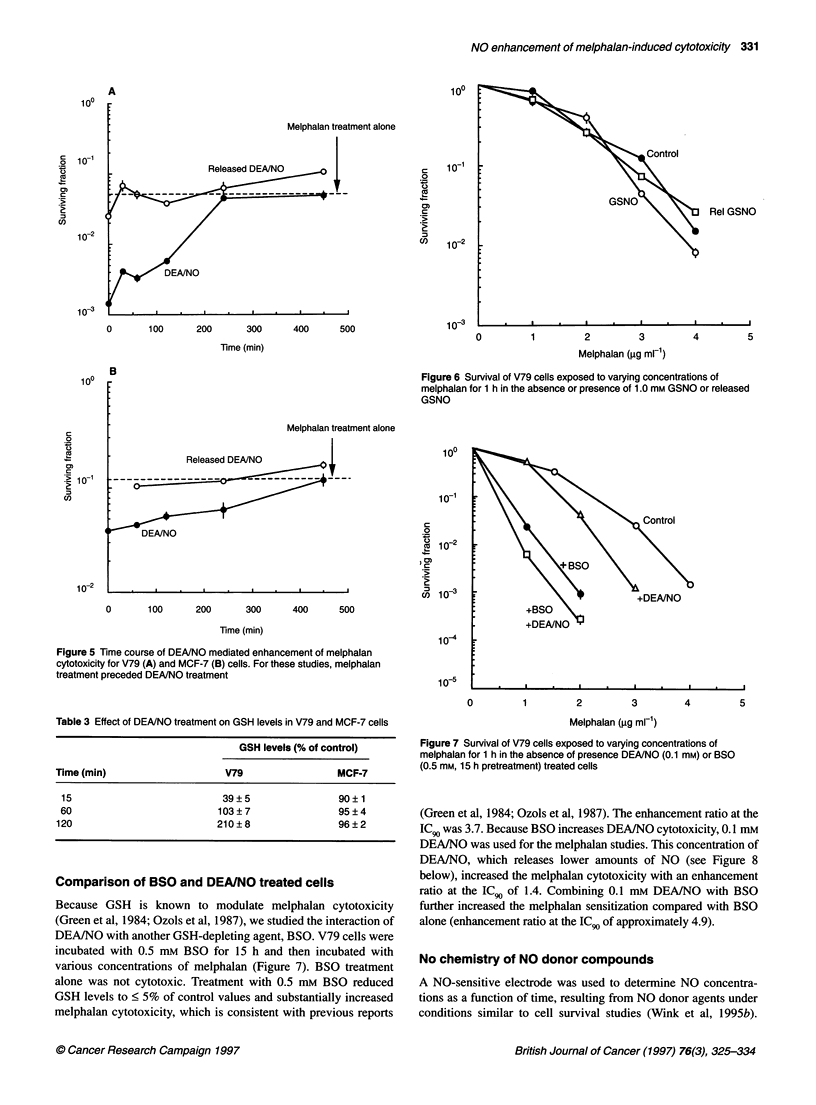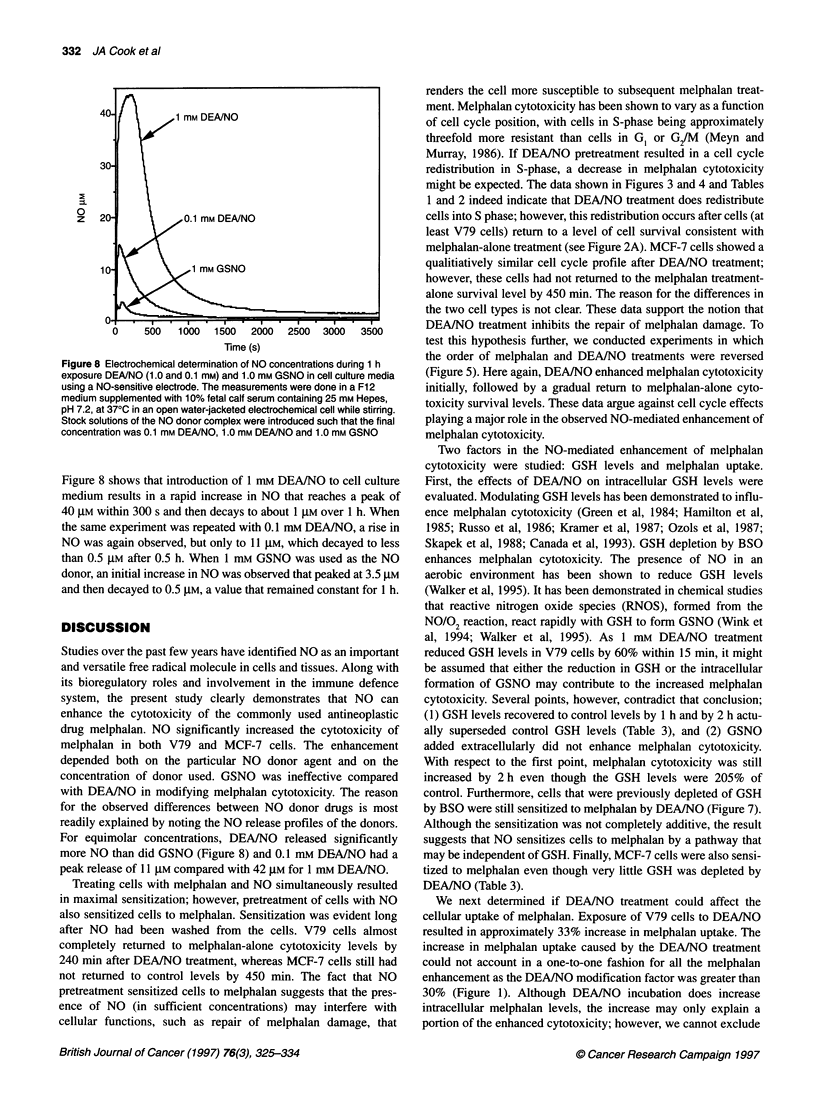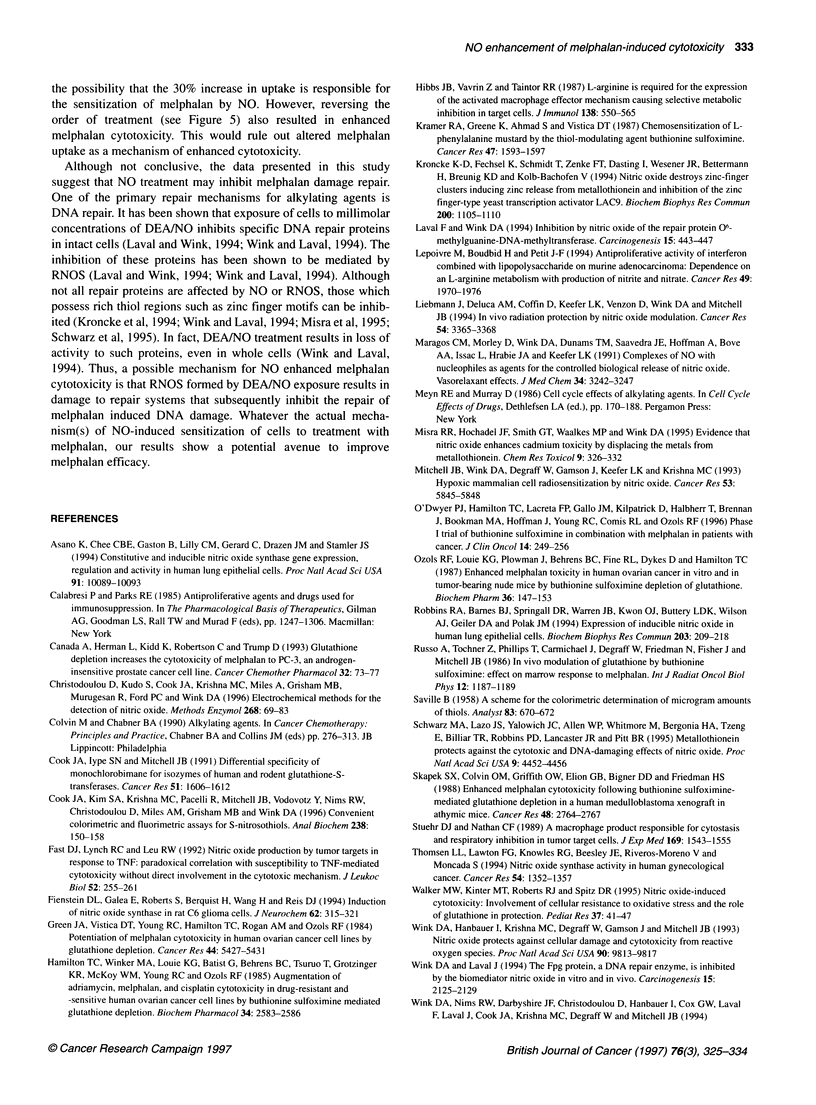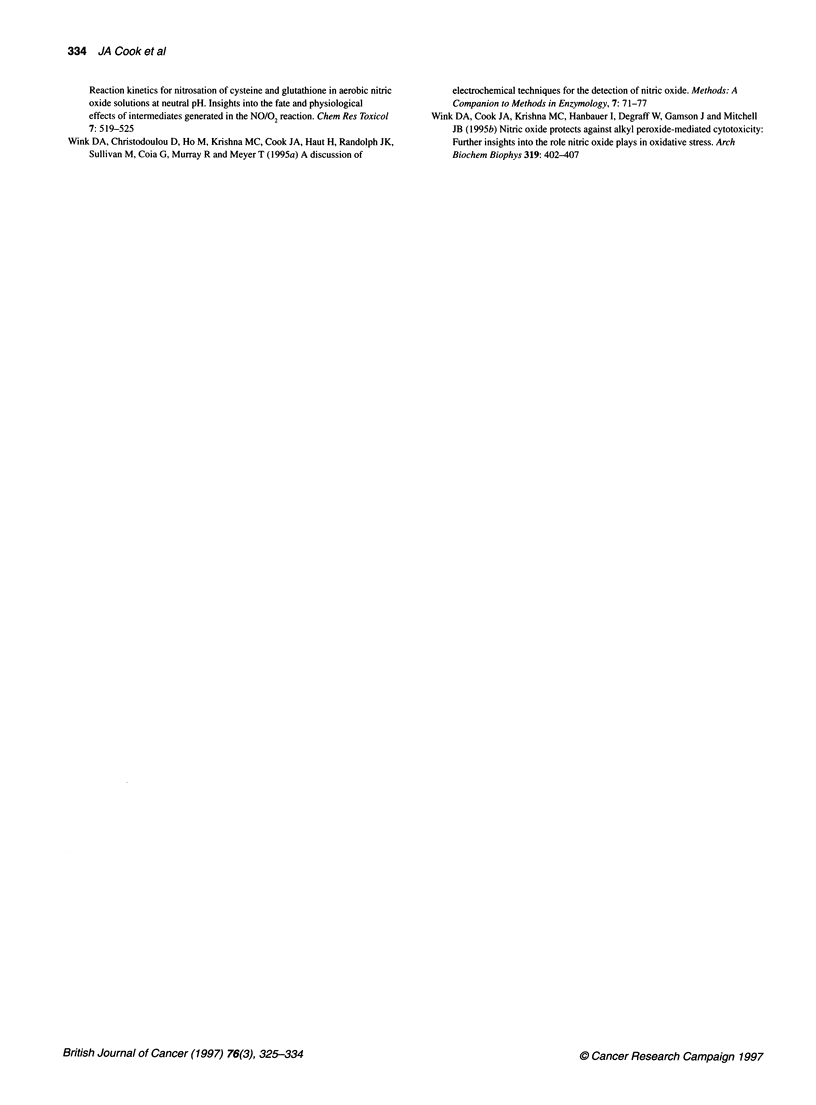# Nitric oxide enhancement of melphalan-induced cytotoxicity.

**DOI:** 10.1038/bjc.1997.386

**Published:** 1997

**Authors:** J. A. Cook, M. C. Krishna, R. Pacelli, W. DeGraff, J. Liebmann, J. B. Mitchell, A. Russo, D. A. Wink

**Affiliations:** Radiation Biology Branch, National Cancer Institute, Bethesda, MD 20892, USA.

## Abstract

The effects of the diatomic radical, nitric oxide (NO), on melphalan-induced cytotoxicity in Chinese hamster V79 and human MCF-7 breast cancer cells were studied using clonogenic assays. NO delivered by the NO-releasing agent (C2H5)2N[N(O)NO]- Na+ (DEA/NO; 1 mM) resulted in enhancement of melphalan-mediated toxicity in Chinese hamster V79 lung fibroblasts and human breast cancer (MCF-7) cells by 3.6- and 4.3-fold, respectively, at the IC50 level. Nitrite/nitrate and diethylamine, the ultimate end products of DEA/NO decomposition, had little effect on melphalan cytotoxicity, which suggests that NO was responsible for the sensitization. Whereas maximal sensitization of melphalan cytotoxicity by DEA/NO was observed for simultaneous exposure of DEA/NO and melphalan, cells pretreated with DEA/NO were sensitized to melphalan for several hours after NO exposure. Reversing the order of treatment also resulted in a time-dependent enhancement in melphalan cytotoxicity. To explore possible mechanisms of NO enhancement of melphalan cytotoxicity, the effects of DEA/NO on three factors that might influence melphalan toxicity were examined, namely NO-mediated cell cycle perturbations, intracellular glutathione (GSH) levels and melphalan uptake. NO pretreatment resulted in a delayed entry into S phase and a G2/M block for both V79 and MCF-7 cells; however, cell cycle redistribution for V79 cells occurred after the cells returned to a level of cell survival, consistent with treatment with melphalan alone. After 15 min exposure of V79 cells to DEA/NO (1 mM), GSH levels were reduced to 40% of control values; however, GSH levels recovered fully after 1 h and were elevated 2 h after DEA/NO incubation. In contrast, DEA/NO (1 mM) incubation did not reduce GSH levels significantly in MCF-7 cells (approximately 10%). Melphalan uptake was increased by 33% after DEA/NO exposure in V79 cells. From these results enhancement of melphalan cytotoxicity mediated by NO appears to be complex and may involve several pathways, including possibly alteration of the repair of melphalan-induced lesions. Our observations may give insights for improving tumour kill with melphalan using either exogenous or possibly endogenous sources of NO.


British Journal of Cancer (1997) 76(3), 325–334
© 1997 Cancer Research Campaign

### Nitric oxide enhancement of melphalan-induced cytotoxicity

JA Cook[1], MC Krishna[1], R Pacelli[1], W DeGraff[1], J Liebmann[2], JB Mitchell[1], A Russo[1] and DA Wink[1]

[1]Radiation Biology Branch, National Cancer Institute, Bethesda, MD 20892 and [2]University of New Mexico Cancer Center, Albuquerque, NM 87131

**Summary** The effects of the diatomic radical, nitric oxide (NO), on melphalan-induced cytotoxicity in Chinese hamster V79 and human MCF-7 breast cancer cells were studied using clonogenic assays. NO delivered by the NO-releasing agent $(C_2H_5)_2N[N(O)NO]^-Na^+$ (DEA/NO; 1 mM) resulted in enhancement of melphalan-mediated toxicity in Chinese hamster V79 lung fibroblasts and human breast cancer (MCF-7) cells by 3.6- and 4.3-fold, respectively, at the $IC_{50}$ level. Nitrite/nitrate and diethylamine, the ultimate end products of DEA/NO decomposition, had little effect on melphalan cytotoxicity, which suggests that NO was responsible for the sensitization. Whereas maximal sensitization of melphalan cytotoxicity by DEA/NO was observed for simultaneous exposure of DEA/NO and melphalan, cells pretreated with DEA/NO were sensitized to melphalan for several hours after NO exposure. Reversing the order of treatment also resulted in a time-dependent enhancement in melphalan cytotoxicity. To explore possible mechanisms of NO enhancement of melphalan cytotoxicity, the effects of DEA/NO on three factors that might influence melphalan toxicity were examined, namely NO-mediated cell cycle perturbations, intracellular glutathione (GSH) levels and melphalan uptake. NO pretreatment resulted in a delayed entry into S phase and a $G_2$/M block for both V79 and MCF-7 cells; however, cell cycle redistribution for V79 cells occurred after the cells returned to a level of cell survival, consistent with treatment with melphalan alone. After 15 min exposure of V79 cells to DEA/NO (1 mM), GSH levels were reduced to 40% of control values; however, GSH levels recovered fully after 1 h and were elevated 2 h after DEA/NO incubation. In contrast, DEA/NO (1 mM) incubation did not reduce GSH levels significantly in MCF-7 cells (approximately 10%). Melphalan uptake was increased by 33% after DEA/NO exposure in V79 cells. From these results enhancement of melphalan cytotoxicity mediated by NO appears to be complex and may involve several pathways, including possibly alteration of the repair of melphalan-induced lesions. Our observations may give insights for improving tumour kill with melphalan using either exogenous or possibly endogenous sources of NO.

Melphalan is a widely used chemotherapeutic agent for a variety of tumour types and is thought to exert its cytotoxic effect by alkylating and cross-linking DNA (Calabresi and Parks, 1985; Colvin and Chabner, 1990). Although melphalan has shown efficacy alone or in combination with other chemotherapeutic agents, there remains a need to explore avenues to broaden its spectrum and effectiveness. Intracellular glutathione (GSH) levels have been shown to modulate melphalan cytotoxicity, and depletion of GSH by buthionine sulphoximine (BSO) has been shown to enhance melphalan cytotoxicity significantly (Green et al, 1984; Ozols et al, 1987). Based on these preclinical findings, phase I clinical trials have been completed recently establishing a safe BSO dose to use in conjunction with melphalan (O'Dwyer et al, 1996).

We have examined the effects of nitric oxide (NO) on the cytotoxicity of various agents and found that NO can modulate the cytotoxicity of various chemicals and ionizing radiation (Mitchell et al, 1993; Wink et al, 1993; Laval and Wink, 1994; Liebmann et al, 1994). NO is an endogenously produced radical that evokes a variety of physiological responses and has been postulated to function in the tumoricidal activity of the immune system (Hibbs et al, 1987; Stuehr and Nathan, 1989). Furthermore, the enzyme nitric oxide synthase (NOS) has been shown to be expressed in various tumours (Asano et al, 1994; Fast et al, 1992; Fienstein et al, 1994; Lepoivre et al, 1994; Robbins et al, 1994; Thomsen et al, 1994), an observation that suggests a potential endogenous source of this agent in vivo.

Because of the recent availability of chemical agents that can release NO (Maragos et al, 1991) and our recent findings that NO can enhance the cytotoxicity of BCNU (Laval and Wink, 1994), we have studied the effect of NO on the cytotoxicity of melphalan in Chinese hamster V79 lung fibroblasts and human breast cancer (MCF-7) cells. We report here that specific NO donor agents can significantly enhance the cytotoxicity of melphalan and may provide an interesting pharmacological approach for improving the clinical efficacy of melphalan.

*Received 8 August 1996*
*Revised 9 January 1997*
*Accepted 17 January 1997*

*Correspondence to:* DA Wink

### MATERIALS AND METHODS

### Chemicals

DEA/NO $\{(C_2H_5)_2N[N(O)NO]^-Na^+\}$ was supplied by Dr J Saavedra and was prepared as previously described (Maragos et al, 1991). *S*-nitrosoglutathione (GSNO) was synthesized as described previously (Saville, 1958). DEA/NO stock solutions were made up in 0.02 N sodium hydroxide and the concentration was determined spectrophotometrically by using an extinction coefficient of 8000 $M^{-1}$ $cm^{-1}$ at its characteristic absorbance band (247 nm) (Mitchell et al, 1993). Hepes (*N*-[2-hydroxyethyl]piperazine-N′-[2-ethanesulphonic acid]) and melphalan were purchased from Sigma (St. Louis, MO, USA). L-buthionine sulphoximine (BSO) was purchased from Schweizerhall (South Plainfield, NJ, USA).

### Cell culture

Chinese hamster V79 lung fibroblasts were cultured in F12 medium supplemented with 10% fetal calf serum and antibiotics. Human breast cancer cells (MCF-7) were obtained from Dr Ken Cowan (Medicine Branch, NCI, NIH, Bethesda, MD, USA) and were grown in RPMI medium (Gibco BRL Gathersburg, MD, USA) supplemented with 10% fetal calf serum and antibiotics. Cell survival was assessed by clonogenic assay; plating efficiencies for V79 and MCF-7 cells were $0.74 \pm 0.1$ and $0.52 \pm 0.06$ respectively. Stock cultures of exponentially growing cells were trypsinized, rinsed and plated ($7 \times 10^5$ cells per dish) into a number of 100-$cm^2$ Petri dishes and incubated for 16 h at 37°C before use in experimental protocols. Cells were exposed to varying concentrations of melphalan (0–4 $\mu g\ ml^{-1}$) for 1 h. Final concentrations of DEA/NO or GSNO (1 mM) were added to parallel cultures immediately before addition of melphalan. In control studies, DEA/NO (1 mM) was allowed to decompose (denoted 'released DEA/NO' on survival curves) in full medium overnight at 37°C before cell exposure and added to parallel cultures immediately before addition of melphalan. GSNO (1 mM) was allowed to decompose (denoted 'released GSNO' on survival curves) in full medium by exposing the solution for 2 h at 365 nm UV (550 kJ $m^{-2}$) (UV Trans-illuminator, UVP, San Gabriel, CA, USA) before cell exposure and added to parallel cultures immediately before addition of melphalan. GSNO decomposure was tested using the RSNO (thiol-nitric oxide) assay described by Cook et al (1996). For studies involving GSH depletion before DEA/NO and/or melphalan treatment, cells were pretreated with 0.5 mM BSO 15 h before experimental procedures as described above. For all treatment conditions, 10 mM Hepes was added to the medium such that the pH was maintained at 7.2. Following treatment, the cells were washed twice with phosphate-buffered saline (PBS), trypsinized, counted and plated for macroscopic colony formation. For each dose determination, cells were plated into triplicate dishes and each experiment was repeated a minimum of two times. Plates were incubated 7 or 10 days (V79, MCF-7 respectively), after which colonies were fixed with methanol–acetic acid (3:1), stained with crystal violet and counted. Colonies containing >50 cells were scored. Error bars represent sd of the mean and are shown when larger than the symbol. Survival curves were corrected for plating efficiency and cytotoxicity for DEA/NO or released DEA/NO where appropriate.

For studies involved in pretreating cells with either DEA/NO or decomposed DEA/NO for 1 h at 37°C and subsequently treating with melphalan, the pretreatment solution was removed and the cells were then rinsed twice with warm complete F12 medium, fresh medium added and then incubated at 37°C. As a function of time after DEA/NO or released DEA/NO treatment, cells were treated with melphalan (final concentration of melphalan for V79 and MCF-7 cells was 1.5 and 2.0 $\mu g\ ml^{-1}$ respectively) for 1 h at 37°C. Following melphalan exposure, cells were plated for survival assessment as described above. Additional studies were conducted in which the order of treatment was reversed.

### Flow cytometry studies

Cells were exposed to 1.0 mM DEA/NO (and released DEA/NO) for 1 h in a fashion identical to cell survival experiments described above. Following treatment, cells were rinsed twice, fresh medium added and incubated at 37°C. At various time intervals after treatment, cells from each treatment group were harvested and fixed in 70% ethanol and stored for DNA analysis. Fixed cells were incubated with 0.4 mg $ml^{-1}$ pepsin (Sigma) in 0.1 N hydrochloric acid for 30 min at 37°C. Cells were spun out of the pepsin and resuspended in 10 mM borate (pH 8.7) to increase the pH and centrifuged immediately. Finally, the samples were resuspended in PBS containing 10 $\mu g\ ml^{-1}$ propidium iodide (PI) to label DNA. Samples were analysed using an EPICS V cell sorter (Coulter Electronics, Hileah, FL, USA) using 488 nm for PI excitation and > 530 nm fluorescence for PI emission. DNA histograms were collected and stored using the CICERO software (Cytomation, Ft Collins, CO, USA). Cell cycle analysis was done using the Multicycle AV DNA/cell cycle analysis software (Phoenix Flow Systems, San Diego, CA, USA). The experiment was repeated twice.

### Glutathione (GSH) measurements

Cells were treated with DEA/NO (1.0 mM) for 60 min at 37°C, rinsed twice, fresh medium added and incubated at 37°C. Control

**Figure 1** Survival of V79 (**A**) and MCF-7 (**B**) cells exposed to varying concentrations of melphalan for 1 h in the absence or presence of 1.0 mM DEA/NO or released DEA/NO

© Cancer Research Campaign 1997

**Figure 2** Time course of DEA/NO mediated enhancement of melphalan cytotoxicity for V79 (**A**) and MCF-7 (**B**) cells. For these studies, DEA/NO treatment preceded melphalan treatment and DEA/NO treated cells were sampled during and after DEA/NO treatment. The samples (in triplicate) were prepared by rinsing the cell monolayer twice with cold PBS and then treating with 0.6% sulphosalicylic acid (4°C) for 5 min. Quadruplicate samples of each condition were assessed for GSH by the monobromobimane–HPLC method (Cook et al, 1991). The total GSH levels for untreated V79 and MCF-7 cells were 1.1 and 31.9 fmol per cell respectively (Cook et al, 1991). Data are expressed as per cent of control values ± s.e.m. and experiments were replicated twice.

### [$^{14}$C]melphalan uptake studies

The effects of DEA/NO or released DEA/NO treatment on V79 cells with respect to melphalan uptake was studied using identical conditions for cell survival procedures as described above. Cells were exposed to radiolabelled melphalan (1.0 μCi $ml^{-1}$, melphalan, [chloroethyl-1,2-$^{14}$C], 50 mCi $mmol^{-1}$, Moravek Biochemicals, Brea, CA, USA) for 1 h in the absence or presence of DEA/NO or released DEA/NO (1 mM, final concentration). Following treatment, cells were rinsed, trypsinized, counted, and aliquots of $10^5$ cells (in triplicate) were placed in scintillation vials containing 10 ml of Ecoscint A (National Diagnostics, Atlanta, GA, USA) and counted in a liquid scintillation counter (Beckman LS6500, Beckman Instruments, Fullerton, CA, USA). Uptake experiments were conducted three times, and the pooled data were analysed by the ANOVA method (two-way analysis of variance).

### NO detection using porphyrin–nafion coated electrode

Electrochemical experiments were conducted with a Princeton Applied Research model 273 potentiostat/galvanostat with PAR 270 software (EG&G Princeton Applied Research, Princeton, NJ, USA). Single carbon fibre electrodes (Medical Systems, Greenvale, NY, USA) having dimensions of 35 μm in diameter and between 100 and 200 μm in length were coated with nickel tetra-*N*-methylpyridiniumporphyrin chloride (Ni(TMPP); MidCentury Chemicals, Posen, IL, USA) as previously described (Wink et al, 1995*a*,*b*). Detection of NO was performed using an amperometric technique at a constant potential of +0.7 V vs saturated calomel electrode (SCE) over a given period of time and standardized with known aliquots of NO as previously described (Wink et al, 1995*a*,*b*). Based on NO-standardized curves, NO concentration values reported are accurate to ± 5%. Measurements of NO derived from the NO donor complexes were conducted in a thermostated cell maintained at 37°C in F12 medium supplemented with 10% fetal calf serum without phenol red. Phenol red interferes with electrochemical detection of NO. Full medium (without cells) was used for these studies to simulate conditions that cells would experience when NO donor agents were added. Experiments were performed by first equilibrating the electrode until the baseline had stabilized; at which point the NO donor was introduced. Experiments were replicated three times. The nickel porphyrin carbon fibre electrode is quite stable and has a sensitivity range of 100 nM NO as previously reported (Christodoulou et al, 1996; Wink et al, 1995*a*).

### RESULTS

### Effect of NO on melphalan cytotoxicity

Treatment of V79 cells with melphalan alone resulted in a concentration-dependent increase in cell killing, as shown in Figure 1A. To determine the effects of NO on melphalan-mediated cytotoxicity, we used a member of the class of compounds known as NONOates (DEA/NO), which spontaneously release NO over a specific time period (Maragos et al, 1991). DEA/NO treatment alone resulted in a 50% reduction in survival for V79 cells. Treatment of cells with 1.0 mM DEA/NO simultaneously with varying concentrations of melphalan resulted in increased melphalan cytotoxicity (Figure 1A). The enhancement in melphalan cytotoxicity by DEA/NO was approximately 3.6- and 2.2-fold at the $IC_{50}$ and $IC_{90}$ levels respectively. Using 0.1 mM DEA/NO, the enhancement factor for an $IC_{90}$ was 1.4 (data shown in Figure 4, see below). In contrast, released DEA/NO, which showed no cytotoxicity to V79 cells alone, did not enhance melphalan cytotoxicity; in fact, modest protection was observed. The use of released DEA/NO was considered an important control because it evaluates if the decomposition products of DEA/NO (namely diethylamine and nitrite) could possibly effect melphalan cytotoxicity. As released DEA/NO did not enhance melphalan cytotoxicity, it can be concluded that NO released from DEA/NO enhanced the melphalan cytotoxicity. Similar findings were found for human MCF-7 cells as shown in Figure 1B. For MCF-7 cells,

**Figure 3** Flow cytometric DNA analysis of V79 cells treated with 1.0 mM DEA/NO and followed as a function of time after treatment

 © Cancer Research Campaign 1997

**Table 1** Cell cycle analysis for V79 cells treated with 1 mM DEA/NO for 1 h

| Time after DEA/NO (h) | $G_1$ (%) | S (%) | $G_2$/M (%) |
|---|---|---|---|
| 0 | 39 | 51 | 10 |
| 1 | 40 | 51 | 9 |
| 2 | 39 | 50 | 11 |
| 3 | 38 | 57 | 5 |
| 4 | 39 | 52 | 9 |
| 5 | 17 | 69 | 14 |
| 6 | 5 | 80 | 15 |
| 7 | 2 | 58 | 40 |

**Table 2** Cell cycle analysis for MCF-7 cells treated with 1 mM DEA/NO for 1 h

| Time after DEA/NO (h) | $G_1$ (%) | S (%) | $G_2$/M (%) |
|---|---|---|---|
| 0 | 29 | 47 | 24 |
| 1 | 30 | 54 | 16 |
| 2 | 23 | 66 | 11 |
| 3 | 30 | 54 | 16 |
| 4 | 34 | 51 | 15 |
| 5 | 24 | 70 | 6 |
| 6 | 25 | 65 | 10 |
| 7 | 20 | 63 | 17 |

DEA/NO treatment also enhanced melphalan cytotoxicity (enhancement at the $IC_{50}$ was approximately 4.3), whereas released DEA/NO had no effect on melphalan cytotoxicity. DEA/NO and released DEA/NO treatment alone both resulted in a 60% reduction in survival for MCF-7 cells.

Studies were next designed to determine the time course of enhanced melphalan cytotoxicity by DEA/NO. For these studies, cells were first treated with DEA/NO or released DEA/NO for 1 h. Following DEA/NO pretreatment, cells were rinsed free of compound and incubated at 37°C in fresh medium. As a function of time after DEA/NO treatment, cells were treated with a fixed concentration of melphalan for 1 h. Figure 2 shows for both V79 and MCF-7 cells that the enhanced melphalan cytotoxicity seen for simultaneous treatment with DEA/NO and melphalan (Figure 1) was also observed following DEA/NO treatment. For V79 cells enhancement of melphalan-induced cell killing by DEA/NO persisted (although not to the extent observed for simultaneous treatment) for at least 30 min, after which there was a steady increase in survival that by 240 min approached that of melphalan treatment alone (Figure 2A). Pretreatment with released DEA/NO did not significantly enhance melphalan cytotoxicity throughout the time course. Figure 2B illustrates qualitatively similar findings for MCF-7 cells. For MCF-7 cells, enhancement of melphalan cytotoxicity was observed throughout the time course studied. Enhancement was greatest from 0 to 120 min after DEA/NO treatment and then there was a gradual increase in survival that came near but never actually reached the melphalan-alone survival level by 450 min. Released DEA/NO treatment did not exert effects on melphalan cytotoxicity (Figure 2B). Collectively, the data in Figures 1 and 2 show that, although melphalan cytotoxicity was enhanced by simultaneous DEA/NO treatment, enhancement could also be observed for considerable time after DEA/NO treatment. With time, the enhancement in melphalan cytotoxicity by DEA/NO pretreatment diminishes, which was variable according to cell type.

Melphalan cytotoxicity has been shown to exhibit cell cycle dependency (Meyn and Murray, 1986). To determine if DEA/NO treatment imposed any perturbations in the cell cycle of V79 or MCF-7 cells that might influence subsequent melphalan cytotoxicity, flow cytometry studies were conducted. Figure 3 shows the DNA flow cytometry results for V79 cells treated with 1 mM DEA/NO for 1 h then washed and followed for times up to 7 h. No major changes in the cell cycle were observed until 5 h, when the cell cycle distributions indicated a delayed entry into S-phase (Figure 3F). This synchronous entry into and through S-phase would indicate that the cells must have been delayed at a specific point at the $G_1$/S border. In addition, $G_1$ continued to decrease and $G_2$/M continued to increase after 5 h (Table 1), which indicates that a $G_2$/M block must also have occurred. Released DEA/NO had no effect on the cell cycle of any time points examined (data not shown). Thus, NO released by DEA/NO has profound cell cycle effects, but these effects do not begin to become evident until 5 h after DEA/NO treatment. The MCF-7 cells treated with DEA/NO responded in a similar fashion. A decrease in $G_1$ and a synchronous increase in S was observed after 7 h; however, the effect was much reduced compared with V79 cells (Figure 4 and Table 2).

Experiments were next conducted where the order of DEA/NO or released DEA/NO and melphalan treatments were reversed. As shown in Figure 5, if melphalan treatment preceded DEA/NO treatment, time-dependent enhancement in melphalan cytotoxicity was observed for both cell lines similar to that shown in Figure 2.

A different type of NO-releasing agent was next evaluated. GSNO, a *S*-nitrosothiol NO donor agent, did not modify melphalan cytotoxicity in V79 cells, as shown in Figure 6. GSNO treatment alone resulted in no cytotoxicity. Likewise, decomposed GSNO was not cytotoxic alone and had no influence on melphalan cytotoxicity.

### Effects of DEA–NO treatment on intracellular GSH levels

As depletion of intracellular GSH levels has been shown to enhance the cytotoxicity of melphalan (Green et al, 1984; Ozols et al, 1987), we conducted experiments to determine if DEA/NO treatment lowered GSH levels. Table 3 shows that a 15-min exposure to DEA/NO reduced GSH levels in V79 cells by approximately 60%; however, after 60 min GSH levels had returned to control levels, and by 120 min there was a substantial increase in GSH levels (205% of control). In contrast, DEA/NO treatment did not significantly alter GSH levels in MCF-7 cells (approximately 10% at 15 min) (Table 3).

### Effects of DEA/NO treatment on melphalan uptake

The enhancement of melphalan cytotoxicity by DEA/NO might result from increased cellular uptake of melphalan. To explore this possibility, V79 cells were treated with radiolabelled melphalan in the absence or presence of 1 mM DEA/NO or released DEA/NO for 1 h (culture conditions identical to cytotoxicity experiments cited above). Melphalan uptake for control, DEA/NO and released DEA/NO treatments was 754 ± 98, 1000 ± 73, and 791 ± 91 d.p.m. respectively. DEA/NO treatment resulted in a significant increase (approximately 33%) in melphalan uptake ($P < 0.01$), whereas released DEA/NO treatment had no significant effect on melphalan uptake.

© Cancer Research Campaign 1997 

**Figure 4** Flow cytometric DNA analysis of MCF-7 cells treated with 1.0 mM DEA/NO and followed as a function of time after treatment

 © Cancer Research Campaign 1997

**Figure 5** Time course of DEA/NO mediated enhancement of melphalan cytotoxicity for V79 (**A**) and MCF-7 (**B**) cells. For these studies, melphalan treatment preceded DEA/NO treatment

**Figure 6** Survival of V79 cells exposed to varying concentrations of melphalan for 1 h in the absence or presence of 1.0 mM GSNO or released GSNO

**Figure 7** Survival of V79 cells exposed to varying concentrations of melphalan for 1 h in the absence of presence DEA/NO (0.1 mM) or BSO (0.5 mM, 15 h pretreatment) treated cells

**Table 3** Effect of DEA/NO treatment on GSH levels in V79 and MCF-7 cells

| | GSH levels (% of control) | |
|---|---|---|
| **Time (min)** | **V79** | **MCF-7** |
| 15 | 39 ± 5 | 90 ± 1 |
| 60 | 103 ± 7 | 95 ± 4 |
| 120 | 210 ± 8 | 96 ± 2 |

### Comparison of BSO and DEA/NO treated cells

Because GSH is known to modulate melphalan cytotoxicity (Green et al, 1984; Ozols et al, 1987), we studied the interaction of DEA/NO with another GSH-depleting agent, BSO. V79 cells were incubated with 0.5 mM BSO for 15 h and then incubated with various concentrations of melphalan (Figure 7). BSO treatment alone was not cytotoxic. Treatment with 0.5 mM BSO reduced GSH levels to ≤ 5% of control values and substantially increased melphalan cytotoxicity, which is consistent with previous reports (Green et al, 1984; Ozols et al, 1987). The enhancement ratio at the $IC_{90}$ was 3.7. Because BSO increases DEA/NO cytotoxicity, 0.1 mM DEA/NO was used for the melphalan studies. This concentration of DEA/NO, which releases lower amounts of NO (see Figure 8 below), increased the melphalan cytotoxicity with an enhancement ratio at the $IC_{90}$ of 1.4. Combining 0.1 mM DEA/NO with BSO further increased the melphalan sensitization compared with BSO alone (enhancement ratio at the $IC_{90}$ of approximately 4.9).

### No chemistry of NO donor compounds

A NO-sensitive electrode was used to determine NO concentrations as a function of time, resulting from NO donor agents under conditions similar to cell survival studies (Wink et al, 1995*b*).

© *Cancer Research Campaign 1997*

**Figure 8** Electrochemical determination of NO concentrations during 1 h exposure DEA/NO (1.0 and 0.1 mM) and 1.0 mM GSNO in cell culture media using a NO-sensitive electrode. The measurements were done in a F12 medium supplemented with 10% fetal calf serum containing 25 mM Hepes, pH 7.2, at 37°C in an open water-jacketed electrochemical cell while stirring. Stock solutions of the NO donor complex were introduced such that the final concentration was 0.1 mM DEA/NO, 1.0 mM DEA/NO and 1.0 mM GSNO

Figure 8 shows that introduction of 1 mM DEA/NO to cell culture medium results in a rapid increase in NO that reaches a peak of 40 μM within 300 s and then decays to about 1 μM over 1 h. When the same experiment was repeated with 0.1 mM DEA/NO, a rise in NO was again observed, but only to 11 μM, which decayed to less than 0.5 μM after 0.5 h. When 1 mM GSNO was used as the NO donor, an initial increase in NO was observed that peaked at 3.5 μM and then decayed to 0.5 μM, a value that remained constant for 1 h.

### DISCUSSION

Studies over the past few years have identified NO as an important and versatile free radical molecule in cells and tissues. Along with its bioregulatory roles and involvement in the immune defence system, the present study clearly demonstrates that NO can enhance the cytotoxicity of the commonly used antineoplastic drug melphalan. NO significantly increased the cytotoxicity of melphalan in both V79 and MCF-7 cells. The enhancement depended both on the particular NO donor agent and on the concentration of donor used. GSNO was ineffective compared with DEA/NO in modifying melphalan cytotoxicity. The reason for the observed differences between NO donor drugs is most readily explained by noting the NO release profiles of the donors. For equimolar concentrations, DEA/NO released significantly more NO than did GSNO (Figure 8) and 0.1 mM DEA/NO had a peak release of 11 μM compared with 42 μM for 1 mM DEA/NO.

Treating cells with melphalan and NO simultaneously resulted in maximal sensitization; however, pretreatment of cells with NO also sensitized cells to melphalan. Sensitization was evident long after NO had been washed from the cells. V79 cells almost completely returned to melphalan-alone cytotoxicity levels by 240 min after DEA/NO treatment, whereas MCF-7 cells still had not returned to control levels by 450 min. The fact that NO pretreatment sensitized cells to melphalan suggests that the presence of NO (in sufficient concentrations) may interfere with cellular functions, such as repair of melphalan damage, that renders the cell more susceptible to subsequent melphalan treatment. Melphalan cytotoxicity has been shown to vary as a function of cell cycle position, with cells in S-phase being approximately threefold more resistant than cells in $G_1$ or $G_2$/M (Meyn and Murray, 1986). If DEA/NO pretreatment resulted in a cell cycle redistribution in S-phase, a decrease in melphalan cytotoxicity might be expected. The data shown in Figures 3 and 4 and Tables 1 and 2 indeed indicate that DEA/NO treatment does redistribute cells into S phase; however, this redistribution occurs after cells (at least V79 cells) return to a level of cell survival consistent with melphalan-alone treatment (see Figure 2A). MCF-7 cells showed a qualitiatively similar cell cycle profile after DEA/NO treatment; however, these cells had not returned to the melphalan treatment-alone survival level by 450 min. The reason for the differences in the two cell types is not clear. These data support the notion that DEA/NO treatment inhibits the repair of melphalan damage. To test this hypothesis further, we conducted experiments in which the order of melphalan and DEA/NO treatments were reversed (Figure 5). Here again, DEA/NO enhanced melphalan cytotoxicity initially, followed by a gradual return to melphalan-alone cytotoxicity survival levels. These data argue against cell cycle effects playing a major role in the observed NO-mediated enhancement of melphalan cytotoxicity.

Two factors in the NO-mediated enhancement of melphalan cytotoxicity were studied: GSH levels and melphalan uptake. First, the effects of DEA/NO on intracellular GSH levels were evaluated. Modulating GSH levels has been demonstrated to influence melphalan cytotoxicity (Green et al, 1984; Hamilton et al, 1985; Russo et al, 1986; Kramer et al, 1987; Ozols et al, 1987; Skapek et al, 1988; Canada et al, 1993). GSH depletion by BSO enhances melphalan cytotoxicity. The presence of NO in an aerobic environment has been shown to reduce GSH levels (Walker et al, 1995). It has been demonstrated in chemical studies that reactive nitrogen oxide species (RNOS), formed from the $NO/O_2$ reaction, react rapidly with GSH to form GSNO (Wink et al, 1994; Walker et al, 1995). As 1 mM DEA/NO treatment reduced GSH levels in V79 cells by 60% within 15 min, it might be assumed that either the reduction in GSH or the intracellular formation of GSNO may contribute to the increased melphalan cytotoxicity. Several points, however, contradict that conclusion; (1) GSH levels recovered to control levels by 1 h and by 2 h actually superseded control GSH levels (Table 3), and (2) GSNO added extracellularly did not enhance melphalan cytotoxicity. With respect to the first point, melphalan cytotoxicity was still increased by 2 h even though the GSH levels were 205% of control. Furthermore, cells that were previously depleted of GSH by BSO were still sensitized to melphalan by DEA/NO (Figure 7). Although the sensitization was not completely additive, the result suggests that NO sensitizes cells to melphalan by a pathway that may be independent of GSH. Finally, MCF-7 cells were also sensitized to melphalan even though very little GSH was depleted by DEA/NO (Table 3).

We next determined if DEA/NO treatment could affect the cellular uptake of melphalan. Exposure of V79 cells to DEA/NO resulted in approximately 33% increase in melphalan uptake. The increase in melphalan uptake caused by the DEA/NO treatment could not account in a one-to-one fashion for all the melphalan enhancement as the DEA/NO modification factor was greater than 30% (Figure 1). Although DEA/NO incubation does increase intracellular melphalan levels, the increase may only explain a portion of the enhanced cytotoxicity; however, we cannot exclude

 © Cancer Research Campaign 1997

the possibility that the 30% increase in uptake is responsible for the sensitization of melphalan by NO. However, reversing the order of treatment (see Figure 5) also resulted in enhanced melphalan cytotoxicity. This would rule out altered melphalan uptake as a mechanism of enhanced cytotoxicity.

Although not conclusive, the data presented in this study suggest that NO treatment may inhibit melphalan damage repair. One of the primary repair mechanisms for alkylating agents is DNA repair. It has been shown that exposure of cells to millimolar concentrations of DEA/NO inhibits specific DNA repair proteins in intact cells (Laval and Wink, 1994; Wink and Laval, 1994). The inhibition of these proteins has been shown to be mediated by RNOS (Laval and Wink, 1994; Wink and Laval, 1994). Although not all repair proteins are affected by NO or RNOS, those which possess rich thiol regions such as zinc finger motifs can be inhibited (Kroncke et al, 1994; Wink and Laval, 1994; Misra et al, 1995; Schwarz et al, 1995). In fact, DEA/NO treatment results in loss of activity to such proteins, even in whole cells (Wink and Laval, 1994). Thus, a possible mechanism for NO enhanced melphalan cytotoxicity is that RNOS formed by DEA/NO exposure results in damage to repair systems that subsequently inhibit the repair of melphalan induced DNA damage. Whatever the actual mechanism(s) of NO-induced sensitization of cells to treatment with melphalan, our results show a potential avenue to improve melphalan efficacy.

### REFERENCES

Asano K, Chee CBE, Gaston B, Lilly CM, Gerard C, Drazen JM and Stamler JS (1994) Constitutive and inducible nitric oxide synthase gene expression, regulation and activity in human lung epithelial cells. *Proc Natl Acad Sci USA* **91**: 10089–10093

Calabresi P and Parks RE (1985) Antiproliferative agents and drugs used for immunosuppression. In *The Pharmacological Basis of Therapeutics*, Gilman AG, Goodman LS, Rall TW and Murad F (eds), pp. 1247–1306. Macmillan: New York

Canada A, Herman L, Kidd K, Robertson C and Trump D (1993) Glutathione depletion increases the cytotoxicity of melphalan to PC-3, an androgen-insensitive prostate cancer cell line. *Cancer Chemother Pharmacol* **32**: 73–77

Christodoulou D, Kudo S, Cook JA, Krishna MC, Miles A, Grisham MB, Murugesan R, Ford PC and Wink DA (1996) Electrochemical methods for the detection of nitric oxide. *Methods Enzymol* **268**: 69–83

Colvin M and Chabner BA (1990) Alkylating agents. In *Cancer Chemotherapy: Principles and Practice*, Chabner BA and Collins JM (eds) pp. 276–313. JB Lippincott: Philadelphia

Cook JA, Iype SN and Mitchell JB (1991) Differential specificity of monochlorobimane for isozymes of human and rodent glutathione-S-transferases. *Cancer Res* **51**: 1606–1612

Cook JA, Kim SA, Krishna MC, Pacelli R, Mitchell JB, Vodovotz Y, Nims RW, Christodoulou D, Miles AM, Grisham MB and Wink DA (1996) Convenient colorimetric and fluorimetric assays for S-nitrosothiols. *Anal Biochem* **238**: 150–158

Fast DJ, Lynch RC and Leu RW (1992) Nitric oxide production by tumor targets in response to TNF: paradoxical correlation with susceptibility to TNF-mediated cytotoxicity without direct involvement in the cytotoxic mechanism. *J Leukoc Biol* **52**: 255–261

Fienstein DL, Galea E, Roberts S, Berquist H, Wang H and Reis DJ (1994) Induction of nitric oxide synthase in rat C6 glioma cells. *J Neurochem* **62**: 315–321

Green JA, Vistica DT, Young RC, Hamilton TC, Rogan AM and Ozols RF (1984) Potentiation of melphalan cytotoxicity in human ovarian cancer cell lines by glutathione depletion. *Cancer Res* **44**: 5427–5431

Hamilton TC, Winker MA, Louie KG, Batist G, Behrens BC, Tsuruo T, Grotzinger KR, McKoy WM, Young RC and Ozols RF (1985) Augmentation of adriamycin, melphalan, and cisplatin cytotoxicity in drug-resistant and -sensitive human ovarian cancer cell lines by buthionine sulfoximine mediated glutathione depletion. *Biochem Pharmacol* **34**: 2583–2586

Hibbs JB, Vavrin Z and Taintor RR (1987) L-arginine is required for the expression of the activated macrophage effector mechanism causing selective metabolic inhibition in target cells. *J Immunol* **138**: 550–565

Kramer RA, Greene K, Ahmad S and Vistica DT (1987) Chemosensitization of L-phenylalanine mustard by the thiol-modulating agent buthionine sulfoximine. *Cancer Res* **47**: 1593–1597

Kroncke K-D, Fechsel K, Schmidt T, Zenke FT, Dasting I, Wesener JR, Bettermann H, Breunig KD and Kolb-Bachofen V (1994) Nitric oxide destroys zinc-finger clusters inducing zinc release from metallothionein and inhibition of the zinc finger-type yeast transcription activator LAC9. *Biochem Biophys Res Commun* **200**: 1105–1110

Laval F and Wink DA (1994) Inhibition by nitric oxide of the repair protein $O^6$-methylguanine-DNA-methyltransferase. *Carcinogenesis* **15**: 443–447

Lepoivre M, Boudbid H and Petit J-F (1994) Antiproliferative activity of interferon combined with lipopolysaccharide on murine adenocarcinoma: Dependence on an L-arginine metabolism with production of nitrite and nitrate. *Cancer Res* **49**: 1970–1976

Liebmann J, Deluca AM, Coffin D, Keefer LK, Venzon D, Wink DA and Mitchell JB (1994) In vivo radiation protection by nitric oxide modulation. *Cancer Res* **54**: 3365–3368

Maragos CM, Morley D, Wink DA, Dunams TM, Saavedra JE, Hoffman A, Bove AA, Issac L, Hrabie JA and Keefer LK (1991) Complexes of NO with nucleophiles as agents for the controlled biological release of nitric oxide. Vasorelaxant effects. *J Med Chem* **34**: 3242–3247

Meyn RE and Murray D (1986) Cell cycle effects of alkylating agents. In *Cell Cycle Effects of Drugs*, Dethlefsen LA (ed.), pp. 170–188. Pergamon Press: New York

Misra RR, Hochadel JF, Smith GT, Waalkes MP and Wink DA (1995) Evidence that nitric oxide enhances cadmium toxicity by displacing the metals from metallothionein. *Chem Res Toxicol* **9**: 326–332

Mitchell JB, Wink DA, Degraff W, Gamson J, Keefer LK and Krishna MC (1993) Hypoxic mammalian cell radiosensitization by nitric oxide. *Cancer Res* **53**: 5845–5848

O'Dwyer PJ, Hamilton TC, Lacreta FP, Gallo JM, Kilpatrick D, Halbherr T, Brennan J, Bookman MA, Hoffman J, Young RC, Comis RL and Ozols RF (1996) Phase I trial of buthionine sulfoximine in combination with melphalan in patients with cancer. *J Clin Oncol* **14**: 249–256

Ozols RF, Louie KG, Plowman J, Behrens BC, Fine RL, Dykes D and Hamilton TC (1987) Enhanced melphalan toxicity in human ovarian cancer in vitro and in tumor-bearing nude mice by buthionine sulfoximine depletion of glutathione. *Biochem Pharm* **36**: 147–153

Robbins RA, Barnes BJ, Springall DR, Warren JB, Kwon OJ, Buttery LDK, Wilson AJ, Geiler DA and Polak JM (1994) Expression of inducible nitric oxide in human lung epithelial cells. *Biochem Biophys Res Commun* **203**: 209–218

Russo A, Tochner Z, Phillips T, Carmichael J, Degraff W, Friedman N, Fisher J and Mitchell JB (1986) In vivo modulation of glutathione by buthionine sulfoximine: effect on marrow response to melphalan. *Int J Radiat Oncol Biol Phys* **12**: 1187–1189

Saville B (1958) A scheme for the colorimetric determination of microgram amounts of thiols. *Analyst* **83**: 670–672

Schwarz MA, Lazo JS, Yalowich JC, Allen WP, Whitmore M, Bergonia HA, Tzeng E, Billiar TR, Robbins PD, Lancaster JR and Pitt BR (1995) Metallothionein protects against the cytotoxic and DNA-damaging effects of nitric oxide. *Proc Natl Acad Sci USA* **9**: 4452–4456

Skapek SX, Colvin OM, Griffith OW, Elion GB, Bigner DD and Friedman HS (1988) Enhanced melphalan cytotoxicity following buthionine sulfoximine-mediated glutathione depletion in a human medulloblastoma xenograft in athymic mice. *Cancer Res* **48**: 2764–2767

Stuehr DJ and Nathan CF (1989) A macrophage product responsible for cytostasis and respiratory inhibition in tumor target cells. *J Exp Med* **169**: 1543–1555

Thomsen LL, Lawton FG, Knowles RG, Beesley JE, Riveros-Moreno V and Moncada S (1994) Nitric oxide synthase activity in human gynecological cancer. *Cancer Res* **54**: 1352–1357

Walker MW, Kinter MT, Roberts RJ and Spitz DR (1995) Nitric oxide-induced cytotoxicity: Involvement of cellular resistance to oxidative stress and the role of glutathione in protection. *Pediat Res* **37**: 41–47

Wink DA, Hanbauer I, Krishna MC, Degraff W, Gamson J and Mitchell JB (1993) Nitric oxide protects against cellular damage and cytotoxicity from reactive oxygen species. *Proc Natl Acad Sci USA* **90**: 9813–9817

Wink DA and Laval J (1994) The Fpg protein, a DNA repair enzyme, is inhibited by the biomediator nitric oxide in vitro and in vivo. *Carcinogenesis* **15**: 2125–2129

Wink DA, Nims RW, Darbyshire JF, Christodoulou D, Hanbauer I, Cox GW, Laval F, Laval J, Cook JA, Krishna MC, Degraff W and Mitchell JB (1994)

© Cancer Research Campaign 1997

Reaction kinetics for nitrosation of cysteine and glutathione in aerobic nitric oxide solutions at neutral pH. Insights into the fate and physiological effects of intermediates generated in the $NO/O_2$ reaction. *Chem Res Toxicol* **7**: 519–525

Wink DA, Christodoulou D, Ho M, Krishna MC, Cook JA, Haut H, Randolph JK, Sullivan M, Coia G, Murray R and Meyer T (1995*a*) A discussion of electrochemical techniques for the detection of nitric oxide. *Methods: A Companion to Methods in Enzymology*, **7**: 71–77

Wink DA, Cook JA, Krishna MC, Hanbauer I, Degraff W, Gamson J and Mitchell JB (1995*b*) Nitric oxide protects against alkyl peroxide-mediated cytotoxicity: Further insights into the role nitric oxide plays in oxidative stress. *Arch Biochem Biophys* **319**: 402–407

© Cancer Research Campaign 1997